# N95 filtering face piece respirators remain effective after extensive reuse during the coronavirus disease 2019 (COVID-19) pandemic

**DOI:** 10.1017/ice.2021.76

**Published:** 2021-02-19

**Authors:** Valeria Fabre, Sara E. Cosgrove, Yea-Jen Hsu, George F. Jones, Taylor Helsel, James Bukowski, Mark Sobota, Anna C. Sick-Samuels, Aaron M. Milstone, Lisa L. Maragakis, Clare Rock

**Affiliations:** 1 Division of Infectious Diseases, Department of Medicine, Johns Hopkins University School of Medicine; 2 Department of Hospital Epidemiology and Infection Control, The Johns Hopkins Hospital; 3 Department of Health Policy and Management, Johns Hopkins Bloomberg of School of Public Health; 4 Johns Hopkins Armstrong Institute for Patient Safety and Quality; 5 Department of Health, Safety & Environment, The Johns Hopkins Hospital; 6 Division of Infectious Diseases, Department of Pediatrics, Johns Hopkins University School of Medicine, Baltimore, Maryland

The coronavirus disease 2019 (COVID-19) pandemic has led to a critical shortage of N95 respirators.^1,2^ One conservation strategy is having healthcare workers (HCWs) reuse their own N95s. Based on a study in a simulated environment, the Centers for Disease Control and Prevention (CDC) suggests limiting the number of reuses to 5 per N95 to ensure an adequate safety margin^3^; however, such an approach likely leads to discarding clinically effective N95s earlier than necessary.^4^ Our goal was to evaluate the effectiveness of reused N95s in a real-world healthcare setting during the COVID-19 pandemic and to identify factors that could be used to proactively identify N95 failure.

## Methods

We conducted a cross-sectional evaluation of reused N95s in July–August 2020 in the emergency department and 5 inpatient units at The Johns Hopkins Hospital (JHH), a 1,162-bed academic hospital in Baltimore, Maryland. JHH recommends N95s for any interaction with confirmed or suspected COVID-19 patients and when performing aerosol-generating procedures for all patients, regardless of SARS-CoV-2 status. To preserve N95 supplies, HCWs reuse their own N95s covered with a face shield until there is concern for structural or functional damage, as determined by visual inspection and a user-performed seal check before every donning. HCWs were given a waist pack in which to store their N95s inside a paper bag. Donning and doffing instructions for HCWs are detailed in Supplementary Table 1 (online). On the day of the N95 assessment, participating HCWs were asked questions about the reuse of their N95s, including number of shifts worked with the current N95 and number of donnings per shift. Questions were answered based on best recall by the HCWs. Following the questionnaire, each N95 had a 3-step assessment: first, an inspection for structural damage; second, a user seal test to assess for air leakage during inhalation and exhalation; and third, a qualitative fit testing with standard saccharin solution aerosol protocol.^5^ Any N95 that failed the seal check or the saccharine fit test was further evaluated with a confirmatory quantitative fit test using the ambient aerosol condensation nuclei counter (PortaCount) protocol,^6,7^ a fit factor result <100 is considered a failure. Due to constraints on N95 supply we used the quantitative method to evaluate only those N95s that failed a screening test because the PortaCount test requires insertion of a probe through the N95, rendering the N95 unusable. At the time of the study, the only available N95 models at our institution were 3M 1860 (dome shaped) and 3M 1870 (duck-bill shaped). HCWs whose N95s failed the fit test were given new N95s. The study was approved by the Johns Hopkins Medicine Institutional Review Board.

### Statistical analysis

The primary outcome was a confirmed N95 failure, defined as failure of 1 or more screening tests followed by failure of the quantitative fit test. Secondary outcomes included factors associated with failure, and accuracy of the user seal check in detecting fit failures. We used the Fisher exact test and the Wilcoxon rank-sum test to evaluate categorical and continuous variables. The relationship between the number of repeated N95 donnings and N95 failure was assessed by Kaplan-Meier curves where survival was considered N95 passing. We conducted a sensitivity analysis including the N95s that failed a screening test (seal check or saccharin fit-test) but did not have a confirmatory PortaCount fit test as failures. Based on data from preliminary observations, with a 95% confidence interval and an error margin of 2, the required sample size was 77 HCWs. A 2-sided *P* value < .05 was considered statistically significant. Analyses were performed using STATA software (2019; StataCorp, College Station, TX).

## Results

**Fig. 1.**
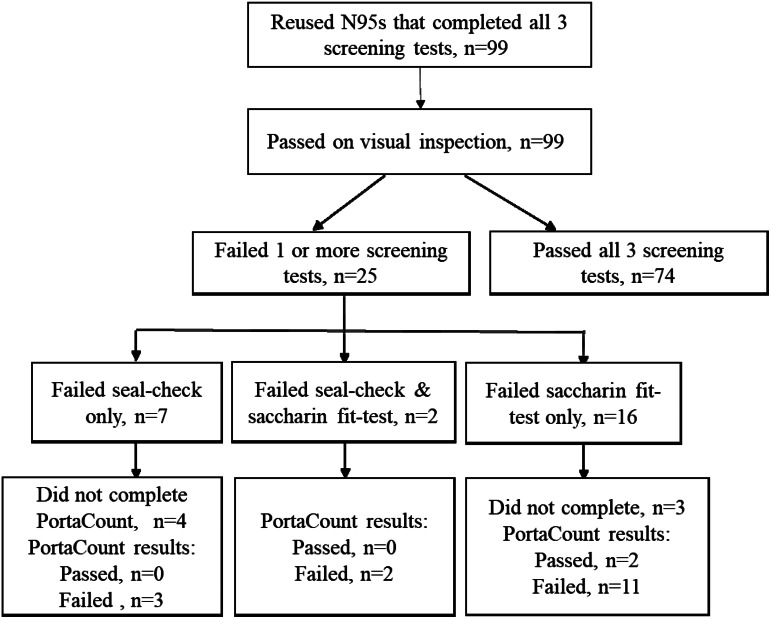
Flow diagram of recruited healthcare workers who reused N95s during the COVID-19 pandemic.

Of 99 HCWs, 92 had complete follow-up (Fig. 1) and were included in the primary analysis. The overall median number of self-reported N95 donnings at the time of the assessment of these 92 HCWs was 40 (interquartile range [IQR], 17–100), and the median for the reported longest number of hours that the N95 was in use once donned was 2.5 hours (IQR, 1–2.5). All 92 N95s were structurally intact upon visual inspection, and 74 (80%) passed both the seal check and the saccharine fit test. Among the 18 N95s that failed 1 or more screening test (Fig. 1), 16 (89%) were confirmed failures with the PortaCount, resulting in an overall 17% N95 failure rate. The N95s of physicians and advanced practitioners were more likely to pass the fit test than the N95s of HCWs in other roles (Table 1).

**Table 1.** Participant Characteristics and Frequency of Repeated N95 Use by Single Healthcare Workers

Cohort Characteristics	All (N = 92)	N95 Pass (N = 76)	N95 Failure (N = 16)	*P* Value
**Sex, no. (%)**				.45
Female	77 (84)	69 (82)	15 (94)	
Male	15 (16)	14 (18)	1 (6)	
**Role, no. (%)**				.01
Physician/Advanced practitioner	22 (24)	21 (28)	1 (6)	
Nurse	54 (59)	44 (58)	10 (63)	
Technician	10 (11)	5 (7)	5 (31)	
Other	6 (6)	6 (8)	0	
**Mask type, no. (%)**				1.00
3M 1860	72 (78%)	59 (78)	13 (81)	
3M 1870	20 (22)	17 (22)	3 (19)	
**N95 use**				
Fold N95 for storage, no. (%)				.47
No	62 (67)	50 (66)	12 (75)	
Yes	30 (33)	26 (34)	4 (25)	
Storage of N95, no. (%)				.30
Waistpack provided by the hospital	65 (71)	55 (73)	10 (62.5)	
Locker	12 (13)	8 (11)	4 (25)	
Bag	14 (15)	12 (16)	2 (12.5)	
Visual Inspection, no. (%)				.65
Not creased	83 (90)	69 (91)	10 (62)	
Creased	9 (10)	7 (9)	6 (38)	
Duration of N95 reuse, no. (%)				.11
	7 (8)	7 (9)	0	
1–4 weeks	33 (36)	24 (32)	9 (56)	
	52 (56)	45 (59)	7 (44)	
N95 donnings, median (IQR)	40 (17.5–100)	50 (20−120)	40 (16−82)	.39
Longest N95 worn once donned, median hours (IQR)	2.5 (1–2.5)	2.5 (1−2.5)	2.5 (1−3.5)	.43

Note. IQR, interquartile range.

Between N95s that did and did not pass the assessment, we detected no differences by 3M N95 type, number of donnings, or reported folding of the N95 during storage. All N95s donned fewer than 12 times passed, and the probability of an N95 maintaining a good fit was >95% for up to 23 donnings (Supplementary Fig. 1 and Supplementary Table 2 online).

The user seal check detected 5 of 16 N95 fit failures (31%).

Cohort characteristics and N95 use factors associated with N95 failure remained similar in a sensitivity analysis that included the 7 HCWs with missing confirmatory PortaCount data as failures. Among these 7 HCWs, only 1 failed the seal check before 12 donnings (Supplementary Table 3 and Supplementary Table 4 online).

## Discussion

This cross-sectional evaluation of HCWs reusing their own 3M N95s until concern for structural or functional damage during the COVID-19 pandemic revealed that 83% were still effective as measured by fit-testing after a median of 40 donnings.

The user seal-check identified 31% of N95s failures. These results demonstrate the value of this simple, noninvasive, quick fit check recommended at each donning to identify gross leakage of air. The minimum number of donnings of N95s that passed the seal check but failed the fit test (covert N95 failures) was 12 times. We estimated the probability of N95s remaining effective at incremental N95 donnings and found that >95% of N95s maintained an adequate fit for up to 23 donnings. Hence, if critical N95 shortages exist, with an effective seal upon donning, HCWs can safely reuse their N95s many times more than the number currently suggested by the CDC.^3^

Even though all N95s were structurally intact upon visual inspection, more N95s in the failure group were noted to be creased. This difference was not statistically significant; however, it may be prudent to advise HCWs to avoid reusing creased masks until they are further evaluated with the saccharin test.

Our study has several limitations. N95s that passed the seal check or the saccharine fit test were not confirmed with the PortaCount due to limited N95 supplies; however, false passes are infrequent with the saccharin method.^8^ We evaluated 2 of the most commonly used N95s in the United States,^9^ and our findings may not be generalizable to alternative models. The number of repeated N95 donnings was based on HCW recall, which may have been under- or over-estimated; however, we do not suspect bias in either direction. N95s were not sampled to assess for pathogen contamination, a risk of N95 reuse, but our face-shield protocol reduces this risk. This study was not powered to assess effectiveness of N95s to prevent COVID-19. Notably, no patient-to-HCW SARS-COV-2 transmissions have been documented for HCWs who complied with the recommended COVID-19 precautions at JHH to date (personal communication). Last, PortaCount data were missing from some HCWs who failed the seal check or the saccharine fit test; however, we performed a sensitivity analysis to minimize the impact of missing data on the interpretation of the study results.

In summary, extensive reuse of the N95 models tested in our study seems an acceptable and safe approach during critical supply shortages rather than uniform discarding of N95s after the currently suggested 5 reuses^3^ as long as HCWs consistently perform a seal check and obtain a good a seal before donning a reused N95. Consideration could be given to offering regular-interval saccharine fit testing when reusing N95s to enhance HCW comfort and safety with respirator reuse.

## Supplementary material

For supplementary material accompanying this paper visit http://dx.doi.org/10.1017/ice.2021.76. click here to view supplementary material
