# Supplementary material for: N95 filtering face piece respirators remain effective after extensive reuse during the coronavirus disease 2019 (COVID-19) pandemic
Source: Infect Control Hosp Epidemiol. 2021 Feb 19:1–4. doi: 10.1017/ice.2021.76 (PMC7971774; doi:10.1017/ice.2021.76)
Supplement: Supplementary file 1 [file S0899823X21000763sup001.docx]

**Supplementary Figure 1:** Probability of N95 maintaining a good fit after incremental donnings.


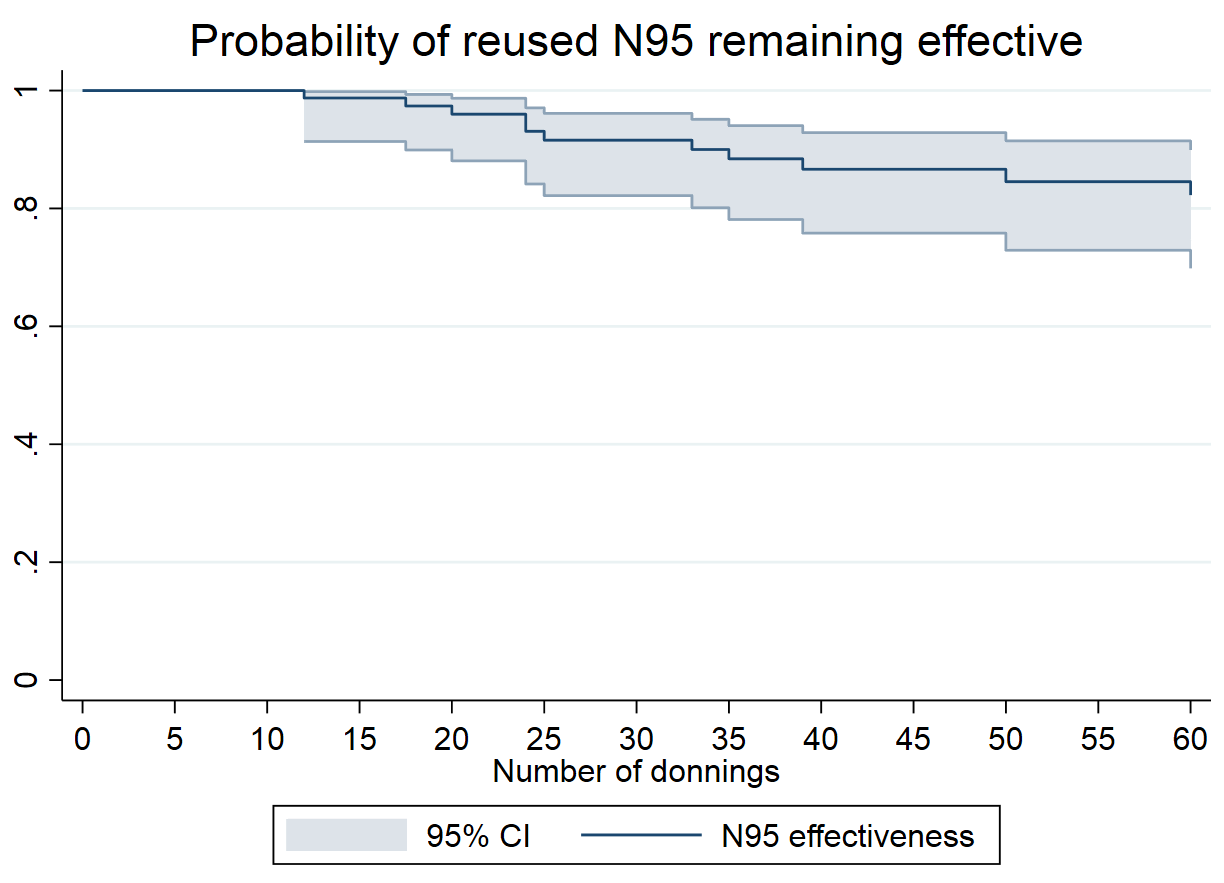


**Supplementray Table 1.** Donning and doffing instructions of personal protective equipment during COVID-19 pandemic.

| **Donning steps** | **Doffing steps** |
| --- | --- |
| Purell hands | Purell outer gloves and remove |
| Don N95 and perform seal check | Remove gown |
| Don inner gloves | Purell inner gloves and leave patient room |
| Don gown | Purell inner gloves again |
| Don face shiled | Remove face shiled and clean |
| Don outer gloves | Remove inner gloves |
|  | Purell hands |
|  | Doff N95 and store until next use |

**Supplementary Table 2:** Probability of N95 maintaining a good fit with incremental donnings.

| **Interval** | **Total** | **Failures per donning interval** | **Pass with no subsequent follow up** | **Passing** | **Error** | **95% Confidence Interval** |
| --- | --- | --- | --- | --- | --- | --- |
| 1 2 | 92 | 0 | 3 | 1 | 0 | . . |
| 3 4 | 89 | 0 | 1 | 1 | 0 | . . |
| 4 5 | 88 | 0 | 1 | 1 | 0 | . . |
| 5 6 | 87 | 0 | 1 | 1 | 0 | . . |
| 7 8 | 86 | 0 | 1 | 1 | 0 | . . |
| 10 11 | 85 | 0 | 6 | 1 | 0 | . . |
| 12 13 | 79 | 1 | 4 | 1 | 0.0129 | 0.9114 0.9982 |
| 15 16 | 74 | 0 | 1 | 0.9870 | 0.0129 | 0.9114 0.9982 |
| 17 18 | 73 | 1 | 2 | 0.9733 | 0.0186 | 0.8974 0.9933 |
| 20 21 | 70 | 1 | 2 | 0.9592 | 0.0231 | 0.8787 0.9867 |
| 22 23 | 67 | 0 | 1 | 0.9592 | 0.0231 | 0.8787 0.9867 |
| 24 25 | 66 | 2 | 2 | 0.9297 | 0.0304 | 0.8390 0.9702 |
| 25 26 | 62 | 1 | 0 | 0.9147 | 0.0334 | 0.8196 0.9608 |
| 28 29 | 61 | 0 | 1 | 0.9147 | 0.0334 | 0.8196 0.9608 |
| 30 31 | 60 | 0 | 2 | 0.9147 | 0.0334 | 0.8196 0.9608 |
| 33 34 | 58 | 1 | 0 | 0.8989 | 0.0363 | 0.7992 0.9506 |
| 35 36 | 57 | 1 | 2 | 0.8829 | 0.0391 | 0.7788 0.9398 |
| 36 37 | 54 | 0 | 3 | 0.8829 | 0.0391 | 0.7788 0.9398 |
| 37 38 | 51 | 0 | 1 | 0.8829 | 0.0391 | 0.7788 0.9398 |
| 39 40 | 50 | 1 | 0 | 0.8652 | 0.0421 | 0.7560 0.9278 |
| 40 41 | 49 | 0 | 2 | 0.8652 | 0.0421 | 0.7560 0.9278 |
| 42 43 | 47 | 0 | 1 | 0.8652 | 0.0421 | 0.7560 0.9278 |
| 45 46 | 46 | 0 | 3 | 0.8652 | 0.0421 | 0.7560 0.9278 |
| 48 49 | 43 | 0 | 1 | 0.8652 | 0.0421 | 0.7560 0.9278 |
| 49 50 | 42 | 0 | 1 | 0.8652 | 0.0421 | 0.7560 0.9278 |
| 50 51 | 41 | 1 | 2 | 0.8436 | 0.0463 | 0.7261 0.9136 |
| 52 53 | 38 | 0 | 1 | 0.8436 | 0.0463 | 0.7261 0.9136 |
| 60 61 | 37 | 1 | 1 | 0.8205 | 0.0504 | 0.6950 0.8980 |
| 63 64 | 35 | 0 | 2 | 0.8205 | 0.0504 | 0.6950 0.8980 |
| 66 67 | 33 | 0 | 1 | 0.8205 | 0.0504 | 0.6950 0.8980 |
| 70 71 | 32 | 0 | 2 | 0.8205 | 0.0504 | 0.6950 0.8980 |
| 73 74 | 30 | 0 | 1 | 0.8205 | 0.0504 | 0.6950 0.8980 |
| 75 76 | 29 | 0 | 1 | 0.8205 | 0.0504 | 0.6950 0.8980 |
| 80 81 | 28 | 0 | 2 | 0.8205 | 0.0504 | 0.6950 0.8980 |
| 84 85 | 26 | 0 | 1 | 0.8205 | 0.0504 | 0.6950 0.8980 |
| 100 101 | 25 | 1 | 0 | 0.7876 | 0.0581 | 0.6456 0.8779 |

**Supplementary Table 3**. Participant characteristics and fit pass rate on a sensitivity analysis where the seven healthcare workers who failed the seal check or the saccharin fit-test and had missing confirmatory PortaCount data are considered failures.

| **Cohort characteristics** | **All, n=99** | **Pass, n = 76** | **Failure, n=23** | ***P* value** |
| --- | --- | --- | --- | --- |
| Gender, n (%) |  |  |  |  |
| Female | 82 | 62 (82) | 20 (87) | 0.54 |
| Male | 17 | 14 (18) | 3 (13) |  |
| Role, n (%) |  |  |  |  |
| Physician/Advanced practitioner | 24 | 21 (28) | 3 (13) | 0.10 |
| Nurse | 56 | 44 (58) | 12 (53) |  |
| Technician | 10 | 5 (50) | 5 (50) |  |
| Other | 9 | 6 (8) | 3 (13) |  |
| Mask type, n (%) |  |  |  |  |
| 3M 1860 | 74 | 59 (78) | 15 (65) | 0.23 |
| 3M 1870 | 25 | 17 (22) | 8 (35) |  |
| **N95 use characteristics** |  |  |  |  |
| Fold N95 for storage, n (%) |  |  |  |  |
| No | 68 | 56 (82) | 12(18) | 0.25 |
| Yes | 31 | 26 (34) | 5 (22) |  |
| User seal check, n (%) |  |  |  |  |
| Fail | 9 | 0 | 9 (39) | <0.01 |
| Pass | 90 | 76 (100) | 14 (61) |  |
| Duration N95 reuse, n (%) |  |  |  |  |
| < 1 week | 9 | 7 (9) | 2 (9) | 0.38 |
| 1-2 weeks | 18 | 11 (14) | 7 (30) |  |
| >2-4 weeks | 16 | 13 (17) | 3 (13) |  |
| > 4 weeks | 56 | 45 (59) | 11 (45) |  |
| Number of repeated donnings, median (IQR) | 43.5 (20-102) | 45 (20−115) | 33 (17.5−60) | 0.28 |
| Longest hours N95 worn after donning, median (IQR) | 2.5 (1-2.5) | 2.5 (1−2.5) | 2.5 (1−2.5) | 0.51 |

IQR: interquartile range.

**Supplementary Table 4:** Probability of passing the fit-test based on 23 failures (in this sensitivity analysis, the seven N95s that failed either the seal check or the saccharin test and that were not confirmed with PortaCount fit-test are considered failures).

| **Interval** | **Total** | **Failures per donning interval** | **Pass with no subsequent follow up** | **Passing** | **Error** | **95% Confidence Interval** |
| --- | --- | --- | --- | --- | --- | --- |
| 1 2 | 99 | 0 | 3 | 1.0000 | 0.0000 | . . |
| 3 4 | 96 | 0 | 1 | 1.0000 | 0.0000 | . . |
| 4 5 | 95 | 0 | 1 | 1.0000 | 0.0000 | . . |
| 5 6 | 94 | 0 | 1 | 1.0000 | 0.0000 | . . |
| 7 8 | 93 | 1 | 1 | 0.9892 | 0.0108 | 0.9257 0.9985 |
| 10 11 | 91 | 0 | 6 | 0.9892 | 0.0108 | 0.9257 0.9985 |
| 11 12 | 85 | 1 | 0 | 0.9776 | 0.0157 | 0.9131 0.9943 |
| 12 13 | 84 | 1 | 4 | 0.9656 | 0.0195 | 0.8971 0.9888 |
| 14 15 | 79 | 1 | 0 | 0.9534 | 0.0228 | 0.8804 0.9823 |
| 15 16 | 78 | 0 | 1 | 0.9534 | 0.0228 | 0.8804 0.9823 |
| 17 18 | 77 | 2 | 2 | 0.9283 | 0.0283 | 0.8471 0.9672 |
| 20 21 | 73 | 1 | 2 | 0.9154 | 0.0307 | 0.8305 0.9588 |
| 21 22 | 70 | 1 | 0 | 0.9023 | 0.0329 | 0.8138 0.9500 |
| 22 23 | 69 | 0 | 1 | 0.9023 | 0.0329 | 0.8138 0.9500 |
| 24 25 | 68 | 2 | 2 | 0.8754 | 0.0370 | 0.7803 0.9311 |
| 25 26 | 64 | 1 | 0 | 0.8617 | 0.0389 | 0.7637 0.9212 |
| 28 29 | 63 | 0 | 1 | 0.8617 | 0.0389 | 0.7637 0.9212 |
| 30 31 | 62 | 0 | 2 | 0.8617 | 0.0389 | 0.7637 0.9212 |
| 33 34 | 60 | 1 | 0 | 0.8474 | 0.0408 | 0.7462 0.9106 |
| 35 36 | 59 | 1 | 2 | 0.8328 | 0.0426 | 0.7286 0.8996 |
| 36 37 | 56 | 0 | 3 | 0.8328 | 0.0426 | 0.7286 0.8996 |
| 37 38 | 53 | 0 | 1 | 0.8328 | 0.0426 | 0.7286 0.8996 |
| 39 40 | 52 | 1 | 0 | 0.8167 | 0.0447 | 0.7089 0.8877 |
| 40 41 | 51 | 0 | 2 | 0.8167 | 0.0447 | 0.7089 0.8877 |
| 42 43 | 49 | 0 | 1 | 0.8167 | 0.0447 | 0.7089 0.8877 |
| 45 46 | 48 | 0 | 3 | 0.8167 | 0.0447 | 0.7089 0.8877 |
| 48 49 | 45 | 0 | 1 | 0.8167 | 0.0447 | 0.7089 0.8877 |
| 49 50 | 44 | 0 | 1 | 0.8167 | 0.0447 | 0.7089 0.8877 |
| 50 51 | 43 | 2 | 2 | 0.7779 | 0.0503 | 0.6595 0.8593 |
| 52 53 | 39 | 0 | 1 | 0.7779 | 0.0503 | 0.6595 0.8593 |
| 56 57 | 38 | 1 | 0 | 0.7574 | 0.0530 | 0.6343 0.8440 |
| 60 61 | 37 | 1 | 1 | 0.7366 | 0.0555 | 0.6093 0.8281 |
| 63 64 | 35 | 0 | 2 | 0.7366 | 0.0555 | 0.6093 0.8281 |
| 66 67 | 33 | 0 | 1 | 0.7366 | 0.0555 | 0.6093 0.8281 |
| 70 71 | 32 | 0 | 2 | 0.7366 | 0.0555 | 0.6093 0.8281 |
| 73 74 | 30 | 0 | 1 | 0.7366 | 0.0555 | 0.6093 0.8281 |
| 75 76 | 29 | 0 | 1 | 0.7366 | 0.0555 | 0.6093 0.8281 |
| 80 81 | 28 | 0 | 2 | 0.7366 | 0.0555 | 0.6093 0.8281 |
| 84 85 | 26 | 0 | 1 | 0.7366 | 0.0555 | 0.6093 0.8281 |
| 100 101 | 25 | 1 | 0 | 0.7072 | 0.0606 | 0.5698 0.8078 |
| 105 106 | 24 | 0 | 1 | 0.7072 | 0.0606 | 0.5698 0.8078 |
| 115 116 | 23 | 0 | 1 | 0.7072 | 0.0606 | 0.5698 0.8078 |
| 120 121 | 22 | 1 | 2 | 0.6735 | 0.0664 | 0.5249 0.7847 |
| 122 123 | 19 | 1 | 1 | 0.6371 | 0.0721 | 0.4784 0.7591 |
| 133 134 | 17 | 0 | 1 | 0.6371 | 0.0721 | 0.4784 0.7591 |
| 156 157 | 16 | 0 | 1 | 0.6371 | 0.0721 | 0.4784 0.7591 |
| 162 163 | 15 | 0 | 1 | 0.6371 | 0.0721 | 0.4784 0.7591 |
| 180 181 | 14 | 1 | 1 | 0.5899 | 0.0807 | 0.4158 0.7280 |
